# Discovery of a peculiar insular race of *Ravenna nivea* (Nire, 1920) (Lepidoptera: Lycaenidae) endemic to Yinggeling Mountain of Hainan, suggesting heterogeneous geological history of mountain formation of the island

**DOI:** 10.7717/peerj.17172

**Published:** 2024-04-23

**Authors:** Yu-Feng Hsu, Yik Fui Philip Lo, Rung-Juen Lin

**Affiliations:** 1 Department of Life Science, National Taiwan Normal University, Taipei, Taiwan, ROC; 2 Kadoorie Conservation China, Kadoorie Farm and Botanic Garden, Tai Po, New Territories, Hong Kong, China; 3 Department of Pediatrics and Medical Genetics, National Taiwan University Hospital, Taipei, Taiwan, ROC

**Keywords:** Zephyrus, Hairstreak, Theclinae, Theclina, Butterfly, Endemism, Glacial relict

## Abstract

A peculiar population of *Ravenna nivea* (Nire, 1920) was discovered from the Yinggeling Mountain Mass of central Hainan. Its wing pattern and COI barcode data show considerable distinction from other geographic populations of *R. nivea*, including that of Bawangling, approximately only 40 km away and also located in Hainan. The p-distance value of the COI barcode between the Yinggeling and Bawangling populations was 1.1%, considerably higher than the value (0.6%) between Bawangling population and populations in eastern China, where the subspecific name *howarthi* Saigusa, 1993 applies. The population is regarded as a distinct subspecies *ngiunmoiae* Lo & Hsu, subsp. nov. The distinctness and high degree of COI haplotype diversity of *R. nivea* found in Hainan and Taiwan suggest continental islands may serve as glacial refugees for the butterfly and other organisms during previous glaciations, and the presence of the relict populations of montane butterflies like *R. nivea* may provide useful clues towards a better understanding of the geological history of mountain formation within islands.

## Introduction

The subspecies concept is one of the most controversial within Linnean taxonomy (Shiaffini, 2020). Although the controversy and utility of the concept has been continuously debated (*e.g.*, Hillis, 2021; de Queiroz, 2021; Burbrink et al., 2022), it is recognized as an available nomen in the species-group category of nominal taxa by International Code of the Zoological Nomenclature (ICZN, 1999), currently in the 4th version. The subspecies concept is widely applied to many groups of organisms, including butterflies for which a scheme for hypothesis testing of taxonomic status of allopatric populations has been proposed (Braby, Eastwood & Murray, 2012). In this scheme, the null hypothesis of a single species (with one or more subspecies) is the default hypothesis and is rejected only if evidence from other multiple data sources (color pattern, morphology, behavior, ecology, genetics, *etc*.) supports the alternative hypothesis of lineage divergence and monophyly. We discovered an intriguing case in lycaenid butterflies in the genus *Ravenna*, in which a population inhabiting two mountain masses separated by a short distance (ca. 40 km) possesses distinct morphological features and genetic make-up. It is suggested that they are each more closely related to different continental populations than to each other, reflecting long isolation since previous glacial period(s).

**Figure 1:**
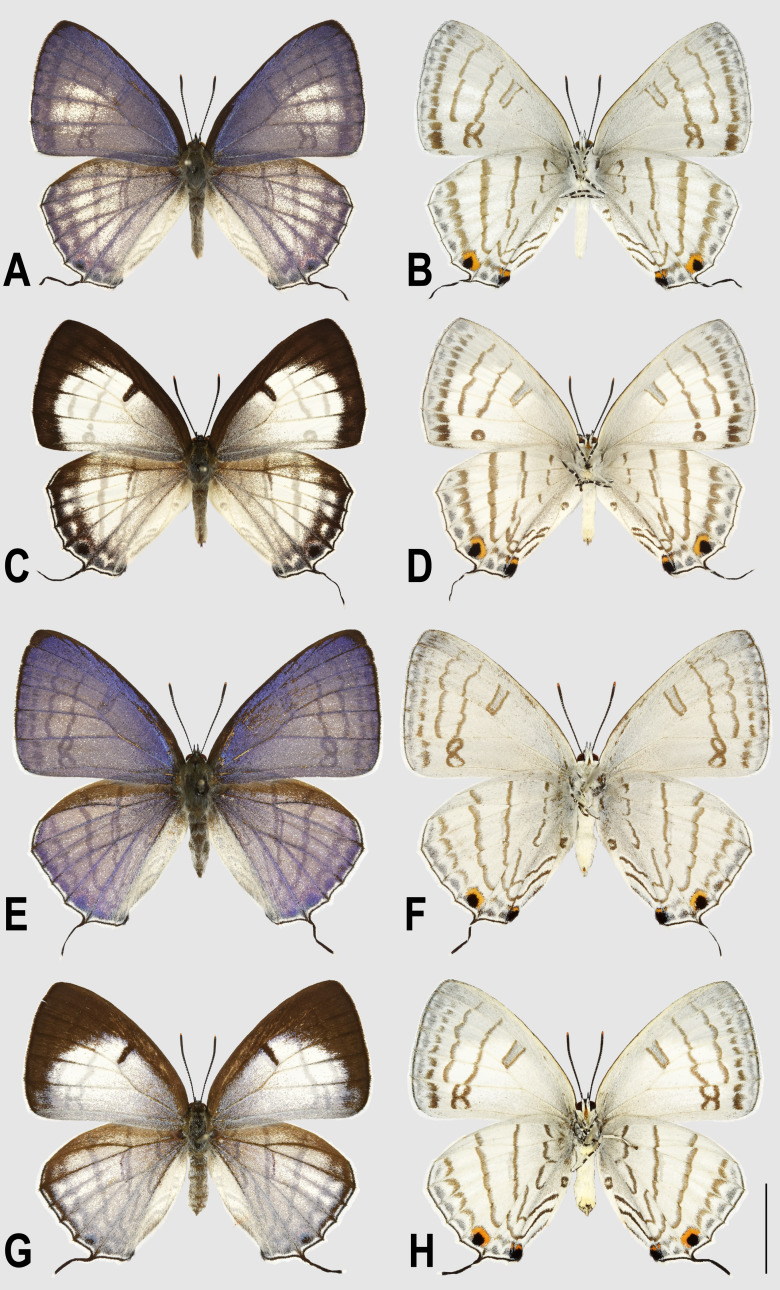
Adults of *Ravenna nivea*. (A) ssp. *howarthi* Saigusa, male, upperside, Bawangling, Hainan. (B) Undersides. (C) Same, female, upperides. (D) Undersides. (E) ssp. *ngiunmoiae* Lo & Hsu, **subsp. nov.**, paratype, male, upperside, Yinggeling, Hainan. (F) Undersides. (G) Same, holotype, female, upperside. (H) Undersides.

The genus *Ravenna* Shirôzu & Yamamoto, 1956 represents a monobasic genus of hairstreak butterflies, containing the sole species *R. nivea* (Nire, 1920). This species inhabits the northern Oriental region, with four subspecies currently recognized: nominotypical *nivea* (Nire, 1920) from Taiwan, *howarthi* Saigusa, 1993 from East to West China, *koiwayai* Yoshino, 1997 from Mt. Konga of Sichuan, and *miyagawai* Katayama & Saito, 2011 from Vietnam. A second species, *R. pacifica* Dubatolov & Korshunov, 1984 was described from Far East Russia but was later transferred to a newly established monobasic genus *Goldia* (Dubatolov & Korshunov, 1990). *Ravenna nivea* is a relatively large species in the tribe Theclini sensu Eliot (1973), usually characterized by the prominent sexual dimorphism of upperside wing pattern, with mostly purple with white markings in males, in contrast to white with distal dark brown margins and markings in females (Figs. 1A–1B). The wing undersides lack sexual dimorphism, with ground color white, decorated with dark brown bands (Figs. 1C–1D). An interesting form, with no white marking on wing upperside of male and purplish scalings extensively overlaid on white area of wing upperside of female (Figs. 1E–1H), was discovered from Yinggeling of central Hainan Island. The peculiar appearance of this form is distinct from populations found elsewhere, calling into question on its appropriate systematic status.

We observed immatures, examined adult genitalia, and sequenced the DNA barcode (COI) of this hairstreak population to determine identity of this hairstreak. Subsequently we came to conclude that this population represents a distinct insular population of *Ravenna nivea* with unique diagnosable features. It is recognized as a new and second insular subspecies of *R. nivea* herein.

## Materials & Methods

### Acquisition of material

Adult butterflies in question were collected from mountain slopes of Yinggeling, Hainan (109°11′27″–109°34′06″, 18°49′30″–19°08′41″), China. Ova were found near dormant buds of the hostplants. Larvae were provided with foliage of the hostplants. Rearing was performed using plastic containers 8 × 5.5 × 3 cm in size, with each larva kept separately.

Besides samples of *Ravenna* collected from Yinggeling, specimens of *R. nivea* from the following localities were examined and compared for morphological characters: ssp. *nivea*, 7♂13♀ (Taiwan); ssp. *howarthi*, 16♂22♀ (2♂2♀, Guangdong, 4♀, Jiangxi, 1♂2♀, Hainan [Bawangling], 1♂1♀, Fujian, 2♂, Zhejiang, 3♂5♀, Sichuan, 7♂8♀, Guizhou); ssp. *miyagawai*, 4♂5♀ (Vietnam). Of them, exemplars of taxa used for sequencing of the COI barcode are given in Table S1. COI barcodes of *Leucantigius atayalicus* and *Yamamotozephyrus kwangtungensis* were obtained from NCBI as reference outgroups (Table S1). Ssp. *koiwayai*, characterized by a reduction of tornal orange markings on hindwing undersides (Yoshino, 1997), is only known from its type locality, Mt. Konga of Sichuan in western China (Koiwaya, 2007), and was not available for the present study. The voucher specimens used for comparison listed above are currently stored in Department of Life Science, National Taiwan Normal University, Taipei (NTNU). The distribution of the samples used in the present study is shown in a map created using the Free and Open Source QGIS. Hainan Wildlife Conservation Bureau, Yinggeling National Nature Reserve and Bawangling National Nature Reserve granted permission to conduct the survey and provided essential assistance with field work.

### Molecular work sampling

Butterfly identification was conducted based on morphological characteristics. The tissue samples were preserved in 95% ethanol and stored frozen at −80 °C for further DNA extraction. Taxa listed in Table S1.

### DNA extraction and sequencing

Tissue from two adult legs was digested using the DNeasy Blood & Tissue kit (Qiagen, Valencia, CA, USA) following the manufacturer’s protocol. The COI gene was amplified by PCR using a set of primers (Table S2). The amplification program was: 5 min at 94 °C, 40 cycles of 30 s at 94 °C, 30 s at 45–50 °C, and 1 min at 72 °C, and a final elongation step of 10 min at 72 °C. The PCR products were run on 1.0% agarose gels in 1x TBE buffer to ensure correct amplification. PCR products were cleaned using a Gel/PCR DNA Fragments Extraction kit (Geneaid, Taipei, Taiwan) when only a single DNA band was visible in a gel. Sequencing reactions were conducted using a 96-well Gel/PCR Clean Up kit (Geneaid) on an ABI3730XL DNA Analyzer (Applied Biosystems, Waltham, MA, USA). All DNA sequences have been submitted to the NCBI GenBank and accession numbers are given in Table S1.

### Molecular data analyses

Molecular sequences of COI gene were checked and assembled into contigs using Sequencher 4.10 (GeneCode, Boston, MA, USA). The software MEGA6 (Tamura, Filipski & Kumar, 2013) was used to perform sequence alignment using the MUSCLE method and sequence divergence using the Kimura2-Parameters (K2P) method. The partition schemes were determined with PartitionFinder v.2.1.1 (Lanfear & Calcott, 2017). The data matrices were analysed using maximum likelihood (ML) with RAxML and Bayesian inference (BI) with MrBayes on CIPRES (Miller, Pfeiffer & Schwartz, 2010). *Leucantigius atayalicus* and *Yamamotozephyrus kwangtungensis* were chosen as the outgroup because the former is considered sister to *Ravenna* by Hsu & Chou (2001) and the latter to be closely related to *Ravenna* by Koiwaya (2007). The program MrBayes 3.2.6 (Ronquist et al., 2012) was used simultaneously for 5 million generations. We removed the first 25% burn-in parts and used the remainder to generate a 50% majority consensus tree. Assessing the effective sample size (ESS), evaluating the parameters, and estimating convergence of two runs were performed with the software Tracer v.1.7 (Rambaut et al., 2018). Phylogenetic trees were read by FigTree v.1.4.3 (see http://tree.bio.ed.ac.uk/software/figtree/). The software program DnaSP 5.10.01 (Rozas et al., 2017) was used to calculate genetic parameters including the nucleotide composition, the number of polymorphic sites, and variable nucleotide positions. A haplotype network was generated *via* median-joining method using POPART 1.7 (Leigh & Bryant, 2015). Haplotype networks were constructed using the TCS analysis in the POPART software.

### Material depository

Primary types are deposited in the following collections: Institute of Zoology, Academia Sinica, Beijing (IOZ), Kadoorie Farm and Botanic Garden, Hong Kong (KFBG), and Department of Biology, National Taiwan Normal University, Taipei (NTNU).

### Terminology for description

Measurements are defined and abbreviated as follows: forewing length (FL) and antennal length (AL). Terminology of wing patterns follows that of Nijhout (1991).

### Nomenclature

The electronic version of this article in Portable Document Format (PDF) will represent a published work according to the International Commission on Zoological Nomenclature (ICZN), and hence the new names contained in the electronic version are effectively published under that Code from the electronic edition alone. This published work and the nomenclatural acts it contains have been registered in ZooBank, the online registration system for the ICZN. The ZooBank LSIDs (Life Science Identifiers) can be resolved and the associated information viewed through any standard web browser by appending the LSID to the prefix http://zoobank.org/. The LSID for this publication is: urn:lsid:zoobank.org:pub:434949A4-F758-4C1D-A332-B2D2E8906098. The online version of this work is archived and available from the following digital repositories: PeerJ, PubMed Central SCIE and CLOCKSS.

## Results

### Genetic divergence

**Figure 2:**
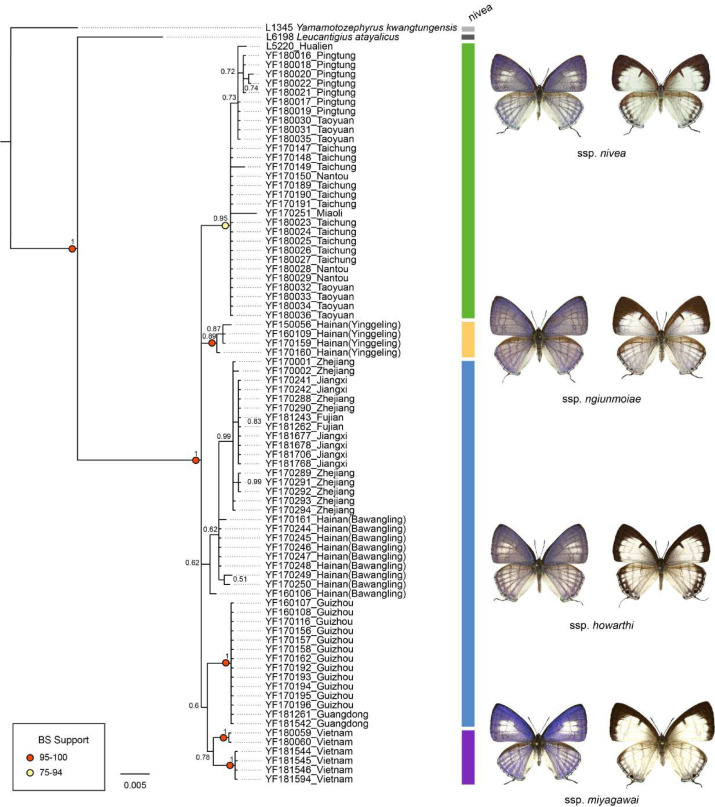
Phylogenetic construction for samples of *Ravenna nivea* from various localities based on the COI barcode resulting from BI analysis. Posterior probabilities shown above branches and bootstrap (BS) value of ML method represented by light yellow or red circles on branches.

**Figure 3:**
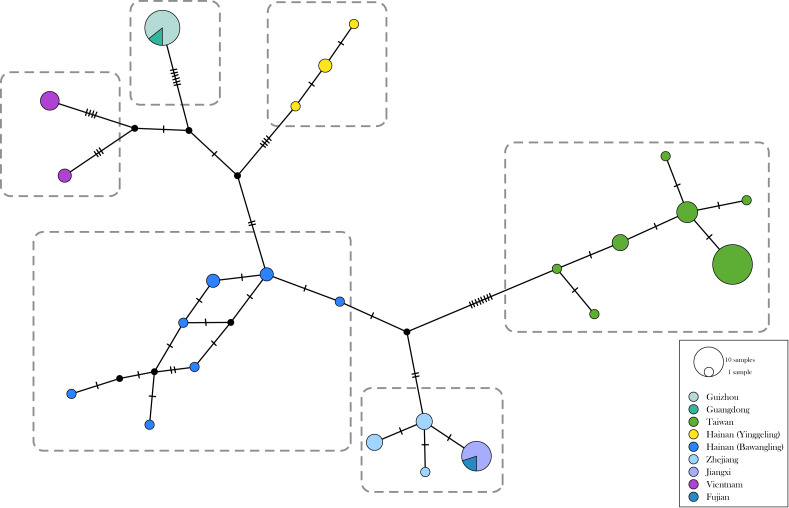
Haplotype networks with haplotype distribution for *Ravenna*. Colors indicate geographic regions. The size of the circle represents the frequency of each haplotype.

The genetic divergence of COI barcode found in the samples used in the present study is shown in Tables S3 and S4. The phylogenetic tree based on BI analysis is given in Fig. 2. The ML tree was slightly different from the BI tree in topology, but all groupings with nodes of bootstrap values over 75% were conformed to the later (Fig. 2). DNA barcode sequences (1,034 bp) were obtained from 80 samples (Table S1). A total of 37 variable sites were detected, with 24 haplotypes defined. Two out of 37 polymorphic sites were singleton variable sites, one from Yinggeling and one from Zhejiang. The variation of mtDNA revealed the presence of 24 haplotypes, seven found only in Taiwan, three restricted to Yinggeling, seven to Bawangling, four to Zhejiang, and two to Vietnam. Samples from Guizhou and Guangdong shared the same haplotype, and those from Jiangxi and Fujian had the same haplotype. The result of the TCS analysis is shown in Fig. 3. The overall haplotype diversity (Hd) was 0.899 and nucleotide diversity (*π*) was 0.012. The values of haplotype diversity and nucleotide diversity that were present are shown in Table S3. The haplotype diversity of *Ravenna nivea* from Yinggeling and Bawangling within Hainan are high, suggesting that the genetic variance is associated with geographic distribution and isolation.

### Taxonomic decision

**Figure 4:**
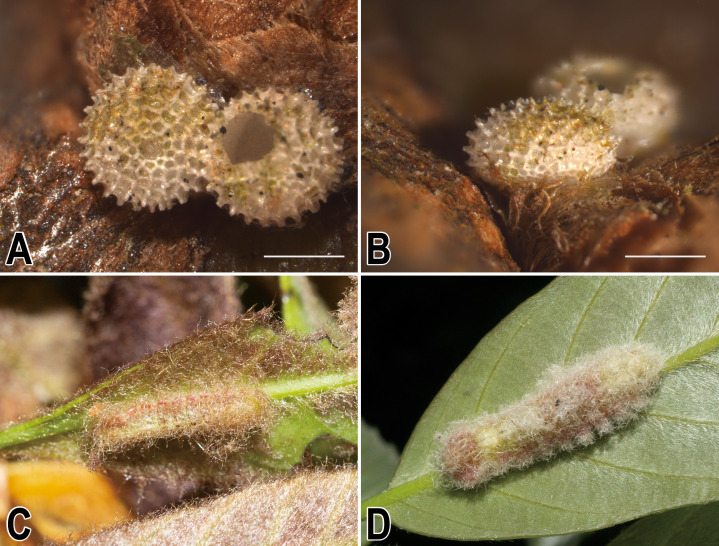
Immatures of *Ravenna nivea ngiunmoiae* Lo & Hsu, subsp. nov. (A) Egg, dorsal view. (B) Egg, lateral view. (C) Larva in 3rd instar. (D) Larva in 4th (final) instar.

**Figure 5:**
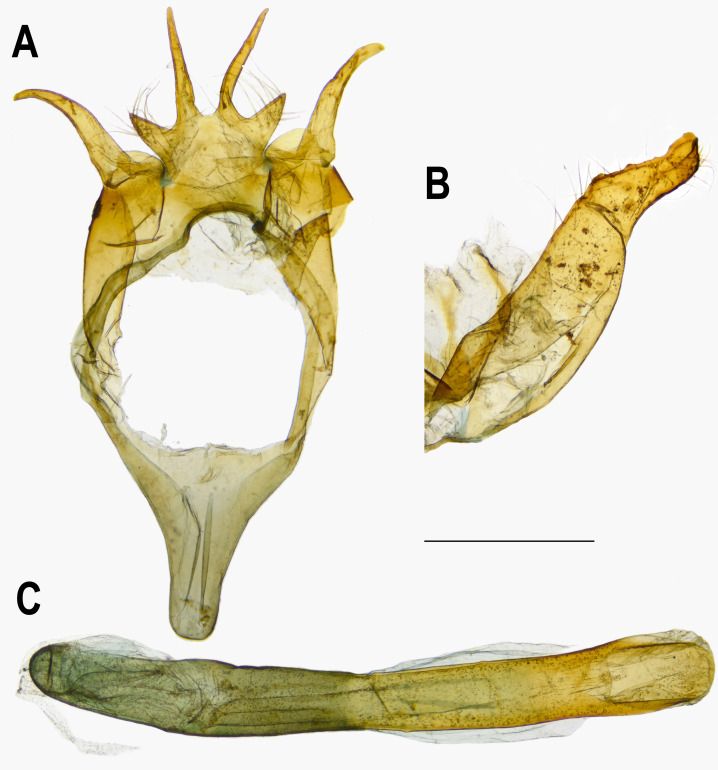
Male genitalia of *Ravenna nivea ngiunmoiae* Lo & Hsu, subsp. nov. (Yinggeling, Hainan). (A) Dorsal view of tegumen. (B) Caudal view of right valva. (C) Ventral view of phallus.

**Figure 6:**
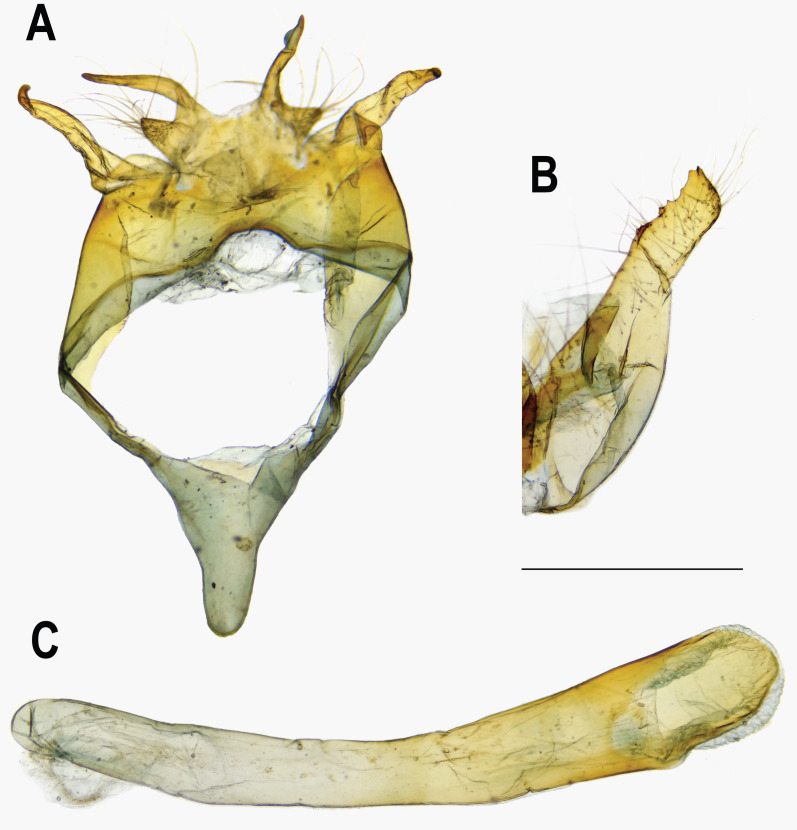
Male genitalia of *Ravenna nivea howarthi* Saigusa (Bawangling, Hainan). (A) Dorsal view of tegumen. (B) Caudal view of right valva. (C) Ventral view of phallus.

**Figure 7:**
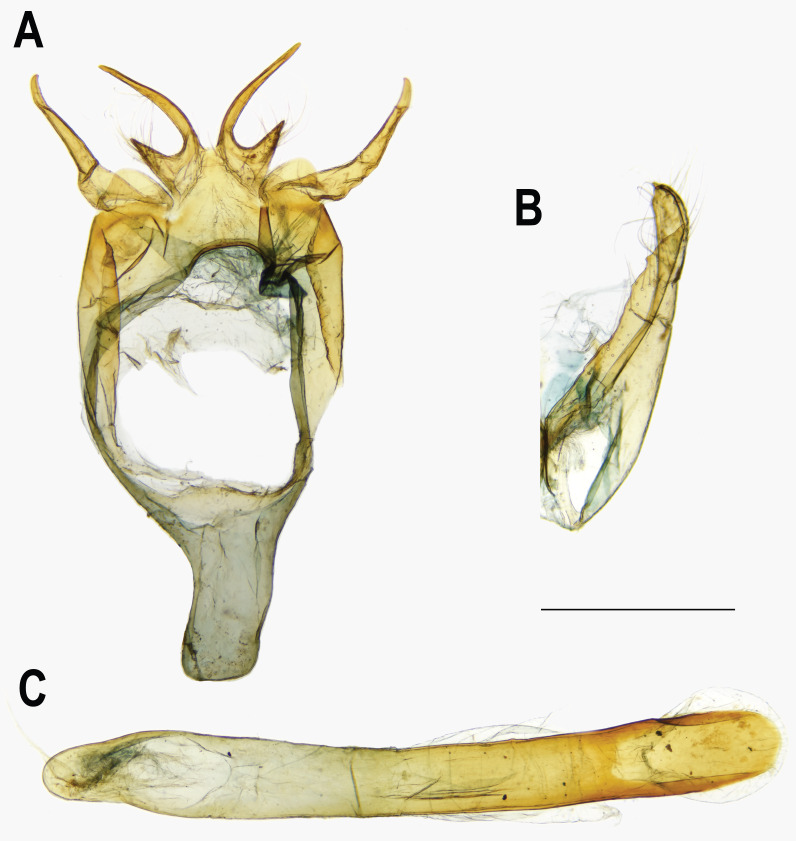
Male genitalia of *Ravenna nivea nivea* Nire (Taiwan). (A) Dorsal view of tegumen. (B) Caudal view of right valva. (C) Ventral view of phallus.

**Figure 8:**
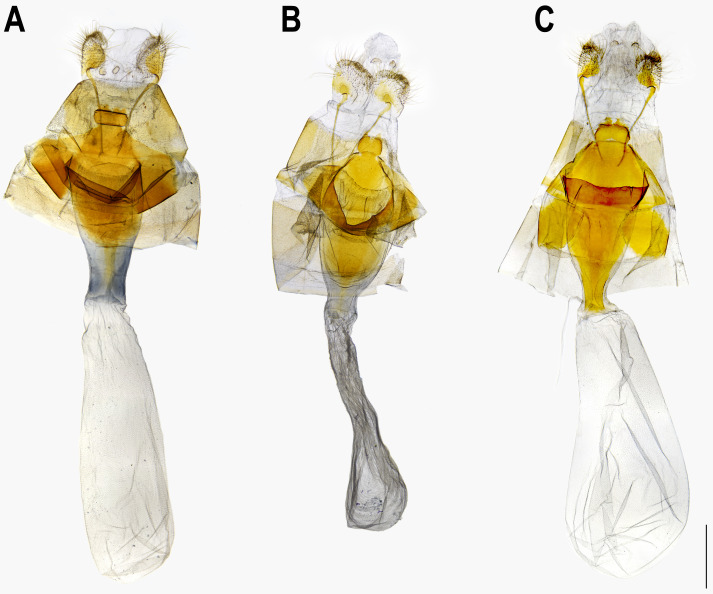
Female genitalia of *Ravenna nivea*. (A) ssp. *ngiunmoiae* Lo & Hsu,** subsp. nov.**(Yinggeling, Hainan). (B) ssp. *howarthi* Saigusa (Guizhou). (C) ssp. *nivea* Nire (Taiwan).

The pairwise genetic divergence of the COI barcode was 0–1.7% between *Ravenna* samples examined from different sites and 1.1–1.5% between the Yinggeling sample and samples from the other localities (Table S4), both of which are lower than the 3% COI genetic divergence for species discrimination of Lepidoptera suggested by Hebert et al. (2003). According to Braby, Eastwood & Murray’s (2012) scheme for testing hypothesis of taxonomic status of allopatric populations, the null hypothesis of a single species is rejected only if evidence from other multiple data sources (color pattern, morphology, behavior, ecology, genetics, *etc*.) supports the alternative. The morphology of immatures of the Yinggeling (Fig. 4) is indistinguishable from that of *R. nivea* from the other localities (see p. 481 in Igarashi & Fukuda, 1997; p. 33 in Uchida, 1999; fig. 45 in Koiwaya, 2007; P. 223 in Lu & Chen, 2014) in morphology and biology. There is also no conceivable diagnosis present in morphology of genitalia of the Yinggeling samples (Figs. 5, 8A) from those of the other subspecies (Figs. 6, 7, 8B, 8C). The morphology, biology, and COI barcodes thus render support to place the Yinggeling *Ravenna* sample within *R. nivea*, but deserving a subspecies status with its well-differentiated wing patterns and genetic make-up. Accordingly, a new subspecies is described herein.

### Systematics

**Table:** 

*Ravenna nivea ngiunmoiae* ** Lo & Hsu, subsp. nov.**
urn:lsid:zoobank.org:act: 418A25B0-63BC-4F3D-872B-C36088CF4A8E
(Figs. 1E–1H, 5, 8A)

**Type materials. Holotype.** ♀: CHINA: Hainan Prov., Baisha Xian, Yinggeling, 1,200–1,300 m, 30. I. 2015, reared from *Qercus fleuryi*, emgd. 4. III. 2015, Coll. Y. F. Hsu & Y. F. Lo (HSU 15A29) (IOZ).

**Paratypes.** 5♀: same locality as for holotype, 1,000–1,400 m, 13. V. 2014, Coll. Y. F. Lo (4♀, KFBG; 1♀, NTNU); 1♂3♀, same collecting data as for holotype, emgd. 26. II. **–**7. III. 2015 (HSU 15A29) (1♂ dissected, genitalia preparation YFH 1565, IOZ; 1♀, NTNU; 2♀, KFBG) (DNA voucher YFH 170160); 1♂, same locality as for holotype, 1,200–1,300 m, 30. I. 2015, reared from *Q. hui*, emgd. 9. III. 2015, Coll. Y. F. Hsu & Y. F. Lo (HSU 15A34) (NTNU); 2♂3♀, same locality as for holotype, 1,200 m, 31. I. 2016, reared from *Q. fleuryi*, emgd. 7–27. III. 2016 (HSU 15A40) (1♂ dissected, genitalia preparation YFH 1561, NTNU; 1♀, NTNU; 1♂2♀, KFBG); 1♂1♀, same locality as for holotype, 1,500 m, 31. I. 2016, reared from *Q. hui*, emgd. 24. III/19. IV. 2016 (HSU 15A50) (NTNU).

**Description.** Male (Figs. 1E–1F): FL 19.2–22.0 mm (mean, 20.4 ± 1.1 mm, *n* = 5); AL 8.3–8.7 mm (mean, 8.6 ± 0.2 mm, *n* = 4). Head: Hairy, vertex, frons dark brown, with mesal white patch on vertex.

Eye semi-oval, sparsely covered with short, buff setae, surrounded by a narrow white rim. Labial palpus porrect, with 3rd segment pointed downwards, covered with white scalings and hairs, dark brown apically. Maxillary palpus reduced, inconspicuous. Proboscis unscaled. Antenna smoothly scaled, naked at terminal end of nudum, dark brown with lateral white dots on each flagellomere. Thorax: Dark brown dorsad, white ventrad. Legs with tarsus of foreleg segmented; white, with brown bands. Forewing: Generally triangular in shape, with apex slightly obtuse; termen, costa slightly convex; dorsum straight. Ground color of upperside purple, with narrow, dark brown margin along termen. Fringe with outer cilia white, inner cilia brown. Ground color of underside white. Discal spot as hollow brown bar. Distal band of central symmetry system represented as a pair of well-separated brown line, approximate in cell Cu_2_. Submarginal band as a series of faint, brown spots; element g (*sensu* Nijhout, 1991) as a broken brown band. Fringe white, prominent. Hindwing: Contour of wing slightly produced at distal end of Cu_1_; Cu_2_ bearing long, tail-like projection. Ground color purple, with brown area along costa; faint, narrow, white margin along termen. Fringe white. Ground color of underside white. Discal spot inconspicuous. Distal band of central symmetry system represented as a pair of brown line, approximate posteriad, dislocated proximally as tick-shaped lines in cell Cu_2_, retracing into straight lines in cell 1A+2A. Proximal band of central symmentry as a broken, brown line. Submarginal band and element g similar to those of forewing, but a black, rounded spot crowned with orange in cell Cu_1_ and at tornus. Fringe white, prominent. Abdomen: Brown dorsally, white ventrally. **Genitalia** (Fig. 5): Ring-shaped sclerites of 9+10 segments with posterior end quadrifurcate. Tegumen broad. Socii prominent, short, conical, setose. Uncus forming a pair of elongate, lateral processes with pointed distal tip, slightly down-curved. Vinculum narrow. Brachia (falces) up-curved, ox horn-like, distal end pointed. Saccus longer than length of tegumen, with anterior end obtuse. Valva elongate, narrowed posteriorly with distal end rounded, small teeth present along inner margin. Juxa broad ventrally, with two arms extending dorsad, forming opposite pointed tips. Phallus with phallobase approximately as long as aedeagus. Aedeagus with dorsal opening at distal end, blunt at caudal tip.

**Female** (Figs. 1G, 1H). FL 19.0–21.7 mm (mean, 20.4 ± 1.1 mm, *n* = 13); AL 7.0–8.8 mm (mean, 8.1 ± 0.6 mm, *n* = 13). Body, wing patterns of underside as described for male except ground color darker. Wing upperside mostly white with extensive purple scalings proximally. Prominent dark brown scalings covering forewing apex, along costa and termen, and along termen of hindwing. Prominent dark brown bar present at distal end of discoidal cell of forewing; faint, narrow, white margin also present along termen of hindwing. **Genitalia** (Fig. 8A): Corpus bursae oval, elongate. Ductus bursae short, thick, sclerotized and enlarged posteriorly, with large ostium bursae. Sterigma with lamella antevaginalis forming a pair of sclerotized, lateral patches; lamella postvaginalis as broad sclerotized wall, with posterior margin produced, blunt. A small, detached transverse, rectangular, sclerotized band posterior to lamella postvaginalis. Posterior apophyses slender, much longer than anal papillae, enlarged and flattened basally. Anal papillae as weakly sclerotized band, setose.

**Immatures.** Egg (Fig. 4A) 0.82 ± 0.03 mm in diameter, 0.49 ± 0.02 mm in height (*n* = 15), hemispherical but compressed, white, surface with sculpture forming framework with regularly arranged, stub-like processes. Larvae (Figs. 4C, 4D) with four instars. Head brown, glossy. Body onisciform, with surface bearing transparent setae; T1 shield as a short, transverse band, slightly produced laterally; anal lobe semi-circular, with posterior margin rounded. Ground color of body green tinged with yellow. Red markings present in late instars, laterally and around medial and caudal portion dorsally. White chevrons present subdorsally. Spiracles white, surrounded by brown peritremes. Full-grown larva (Fig. 4D) reaching 18 mm in body length. Pupae reaching 12 mm in length, of typical lycaenid form, ground color pale brown, decorated with yellow chevrons and dark brown, subdorsal bands; spiracles cream white.

**Diagnosis.** The taxon *ngiunmoiae* **ssp. nov.** can be distinguished from other subspecies of *R. nivea* by the following external features: (1) in the males, prominent white scalings are absent on wing uppersides of *ngiunmoiae* (Fig. 5), whereas present -in the other subspecies (Fig. 1), but; (2) in the females, extensive purple scalings are present on wing upperside in *ngiunmoiae* (Fig. 7), whereas weakly-developed brown scalings are found proximally in the other subspecies (Fig. 3).

**Hostplants.** *Quercus hui* and *Q. fleuryi* (Fagaceae).

**Distribution.** Known from montane zone of Mt. Yinggeling, central Hainan approximately 1,000–1,500 m in elevation.

**Phenology.** Univoltine. Adults were collected in May, suggesting occurrence of adult imago may be in early summer. Overwintering as egg near bases of dormant buds on the hostplants.

**Etymology.** The subspecific name *ngiunmoiae* refers to the pronunciation of the name of the late grandmother 
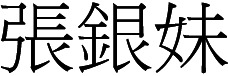
 , in the Hakka dialect, of the first author of the new subspecies, YFP Lo, for her warm and full support to his scientific work on butterflies and other animals.

## Discussion

**Figure 9:**
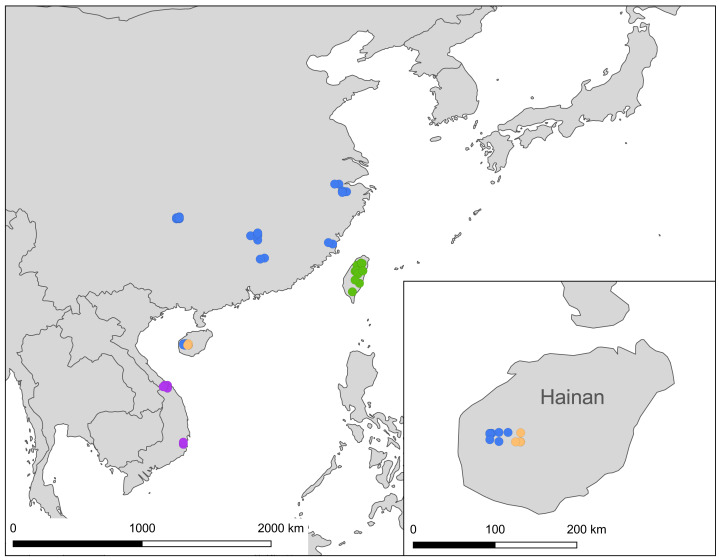
A map showing localities of the *Ravenna nivea* samples used in the present study, with each subspecies depicted by a circle colored in accordance with those shown in Fig. 2.

The distribution of samples used in the present study, with each subspecies depicted by different color, is shown on a map (Fig. 9). The most interesting finding about *Ravenna nivea ngiunmoiae* ssp. nov. is that its type locality Yinggeling is only approximately 40 km distant from Bawangling, where another population of *R. nivea* occurs, with wing patterns indistinguishable from continental race ssp. *howarthi*. It appears that the p-distance of the COI barcode between samples from Bawangling and localities from southern China (Jiangxi, Zhejiang and Fujian) was 0.6%, evidently lower than the value between samples of Yinggeling and Bawangling (1.1%) (Table S4). A phylogenetic analysis also suggested that the population of *R. nivea* from Bawangling is more closely related to those of southern China than to the population of Yinggeling (*ngiunmoiae* ssp. nov.) (Fig. 2). These results suggest that the population of Yinggeling may have been separated from that of Bawangling for a long time, and they may have been established independently from different continental sources, instead of differentiated from a common ancestral source. Geological evidence has shown that Hainan was part of continental China until the early Quaternary, but separated when the Qiongzhou Strait formed, and then subsequently re-connected and re-disconnected due to the arrival and departure of glacial periods (Yan, 2006; Zhao, Wang & Yuan, 2007). Zhu (2016) conducted a biogeographical study based on botanical evidence and suggested that Hainan was closer to Guangxi and Vietnam in Eocene. Nevertheless, the formation history of mountain ranges within Hainan has yet to be established, thus the pathways of immigration for Yinggeling and Bawangling remain unsolved. The fact that the values of haplotype diversity of *Ravenna nivea* found in Taiwan and Yinggeling and Bawangling of Hainan are higher than those in the continental populations (Table S4) suggest islands may serve as glacial refugia for this montane butterfly during glacial periods. Although the value of subspecies concept has been debated, and some authors downplay the utility of subspecies (Burbrink et al., 2022), the case found in the *Ravenna* butterfly demonstrates that the subspecies concept provides clues towards the nature of heterogeneous geological history of mountain formation within Hainan island.

## Conclusions

The subspecies concept has been controversial and criticized for its utility in Linnean taxonomy, but it remains a valid species-group name category in the International Code of the Zoological Nomenclature (ICZN), currently in the 4th version. The discovery of a population of the lycaenid butterfly *Ravenna nivea* inhabiting Yinggeling on Hainan island, unique in genetic make-up and wing pattern, suggests that the subspecies concept may help bring attention to this local population with unique history. Without adoption of the subspecies concept, the significance of such a population to conservation may be overlooked.

## Supplemental Information

10.7717/peerj.17172/supp-1 Supplemental Information 1 Specimens of *Ravenna* hairstreaks used for sequencing of COI barcode in the present study Names of taxa adopt those prior to taxonomic decisions.

10.7717/peerj.17172/supp-2 Supplemental Information 2 Primers used in the present study

10.7717/peerj.17172/supp-3 Supplemental Information 3 Genetic diversity of different populations The number of samples (n), the number of segregating sites (s), the number of haplotypes (h), the haplotype diversity (Hd), and the nucleotide diversity (*π*).

10.7717/peerj.17172/supp-4 Supplemental Information 4 The p-distance of COI sequences between *Ravenna* taxa used in the present study

## Additional Information and Declarations

### DNA Deposition

The following information was supplied regarding the deposition of DNA sequences: The COI gene sequences are available at GenBank: OR667038 to OR667119.

### Data Availability

The following information was supplied regarding data availability: The raw measurements are available in the Supplementary Files.

### New Species Registration

The following information was supplied regarding the registration of a newly described species: *Ravenna nivea ngiunmoiae* Lo & Hsu: urn:lsid:zoobank.org:act: 418A25B0-63BC-4F3D-872B-C36088CF4A8E Publication LSID: urn:lsid:zoobank.org:pub:434949A4-F758-4C1D-A332-B2D2E8906098.
