# Supplementary material for: Discovery of a peculiar insular race of Ravenna nivea (Nire, 1920) (Lepidoptera: Lycaenidae) endemic to Yinggeling Mountain of Hainan, suggesting heterogeneous geological history of mountain formation of the island
Source: PeerJ. 2024 Apr 23;12:e17172. doi: 10.7717/peerj.17172 (PMC11048081; doi:10.7717/peerj.17172)
Supplement: Supplemental Information 2 [file peerj-12-17172-s002.docx]

Table S2. Primers used in the present study.

| Oligo ID | Primer sequence | Direction | Position |
| --- | --- | --- | --- |
| cox-J-1460 | TACAA TTTAT CGCCT AAACT TCAGC C | J | 1460 |
| Zcox-J-1530 | CAACA AATCA TAAAG ATATT GG | J | 1530 |
| MiBocox-J-1700 | AATAC TATTG TTACA GCTCA TGC | J | 1700 |
| Jpcox-J-2040 | CTTTA CCTGT ATTAG CAGGT GC | J | 2040 |
| MiBocox-N-2010 | AGTTG TAATA AAATT AATWG CTCCT A | N | 2010 |
| cox-N-2191 | CCCGG TAAAA TTAAA ATATA AACTT C | N | 2191 |
| MiBocox-N-2450 | GTATC AATAT CTATA CCTAC TGT | N | 2450 |
| Zcox-N-2530 | CTCCT GTTAA TCCTC CTACA GT | N | 2530 |
| MiBocox-N-2860 | TGAAA ATGAG CAACA ACATA ATA | N | 2860 |
