# Supplementary material for: Discovery of a peculiar insular race of Ravenna nivea (Nire, 1920) (Lepidoptera: Lycaenidae) endemic to Yinggeling Mountain of Hainan, suggesting heterogeneous geological history of mountain formation of the island
Source: PeerJ. 2024 Apr 23;12:e17172. doi: 10.7717/peerj.17172 (PMC11048081; doi:10.7717/peerj.17172)
Supplement: Supplemental Information 3 — The number of samples (n), the number of segregating sites (s), the number of haplotypes (h), the haplotype diversity (Hd), and the nucleotide diversity (π). [file peerj-12-17172-s003.docx]

Table S3. Genetic diversity of different populations. The number of samples (n), the number of segregating sites (s), the number of haplotypes (h), the haplotype diversity (Hd), and the nucleotide diversity (π).

| Location | n | s | h | Hd | π |
| --- | --- | --- | --- | --- | --- |
| Taiwan | 30 | 5 | 7 | 0.618 | 0.00140 |
| Yinggeling, Hainan | 4 | 2 | 3 | 0.833 | 0.00127 |
| Bawangling, Hainan | 9 | 6 | 7 | 0.944 | 0.00316 |
| Guizhou | 12 | 0 | 1 | 0.000 | 0.00000 |
| Zhejiang | 9 | 3 | 4 | 0.806 | 0.00141 |
| Jiangxi | 6 | 0 | 1 | 0.000 | 0.00000 |
| Vietnam | 6 | 7 | 2 | 0.533 | 0.00473 |
| Fujian | 2 | 0 | 1 | 0.000 | 0.00000 |
| Guangdong | 2 | 0 | 1 | 0.000 | 0.00000 |
| Total | 80 | 37 | 24 | 0.899 | 0.01160 |
