# Supplementary material for: Discovery of a peculiar insular race of Ravenna nivea (Nire, 1920) (Lepidoptera: Lycaenidae) endemic to Yinggeling Mountain of Hainan, suggesting heterogeneous geological history of mountain formation of the island
Source: PeerJ. 2024 Apr 23;12:e17172. doi: 10.7717/peerj.17172 (PMC11048081; doi:10.7717/peerj.17172)
Supplement: Supplemental Information 4 [file peerj-12-17172-s004.docx]

Table S4. The *p*-distance of COI sequences between *Ravenna* taxa used in the present study.

|  | Taiwan | Yinggeling | Bawangling | Guizhou | Zhejiang | Jiangxi | Vietnam | Fujian | Guangdong |
| --- | --- | --- | --- | --- | --- | --- | --- | --- | --- |
| Taiwan |  |  |  |  |  |  |  |  |  |
| Yinggeling, Hainan | 0.016 |  |  |  |  |  |  |  |  |
| Bawangling, Hainan | 0.016 | 0.011 |  |  |  |  |  |  |  |
| Guizhou | 0.017 | 0.015 | 0.012 |  |  |  |  |  |  |
| Zhejiang | 0.016 | 0.013 | 0.006 | 0.013 |  |  |  |  |  |
| Jiangxi | 0.016 | 0.013 | 0.006 | 0.013 | 0.001 |  |  |  |  |
| Vietnam | 0.017 | 0.012 | 0.012 | 0.013 | 0.014 | 0.014 |  |  |  |
| Fujian | 0.016 | 0.013 | 0.006 | 0.013 | 0.001 | 0.000 | 0.014 |  |  |
| Guangdong | 0.017 | 0.015 | 0.011 | 0.000 | 0.012 | 0.013 | 0.013 | 0.013 |  |
